# Towards structured output prediction of enzyme function

**DOI:** 10.1186/1753-6561-2-s4-s2

**Published:** 2008-12-17

**Authors:** Katja Astikainen, Liisa Holm, Esa Pitkänen, Sandor Szedmak, Juho Rousu

**Affiliations:** 1 Department of Computer Science, PO Box 68, FI-00014 University of Helsinki, Finland; 2 Institute of Biotechnology, P.O. Box 56, FI-00014 University of Helsinki, Finland; 3 Electronics and Computer Science, University of Southampton, SO17 1BJ, UK

## Abstract

**Background:**

In this paper we describe work in progress in developing kernel methods for enzyme function prediction. Our focus is in developing so called structured output prediction methods, where the enzymatic reaction is the combinatorial target object for prediction. We compared two structured output prediction methods, the Hierarchical Max-Margin Markov algorithm (HM^3^) and the Maximum Margin Regression algorithm (MMR) in hierarchical classification of enzyme function. As sequence features we use various string kernels and the GTG feature set derived from the global alignment trace graph of protein sequences.

**Results:**

In our experiments, in predicting enzyme EC classification we obtain over 85% accuracy (predicting the four digit EC code) and over 91% microlabel F1 score (predicting individual EC digits). In predicting the Gold Standard enzyme families, we obtain over 79% accuracy (predicting family correctly) and over 89% microlabel F1 score (predicting superfamilies and families). In the latter case, structured output methods are significantly more accurate than nearest neighbor classifier. A polynomial kernel over the GTG feature set turned out to be a prerequisite for accurate function prediction. Combining GTG with string kernels boosted accuracy slightly in the case of EC class prediction.

**Conclusion:**

Structured output prediction with GTG features is shown to be computationally feasible and to have accuracy on par with state-of-the-art approaches in enzyme function prediction.

## Background

**Figure 1:**
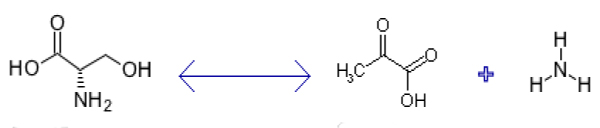
A chemical reaction catalyzed by the enzyme serine deaminase.

Enzymes are the workhorses of living cells, producing energy and building blocks for cell growth as well as participating in maintaining and regulation of the metabolic states of the cells. Reliable assignment of enzyme function, that is, the biochemical reactions (Fig. 1) catalyzed by the enzymes, is a prerequisite of high-quality metabolic reconstruction and the analysis of metabolic fluxes [1].

Protein function taxonomies such as Gene ontology [2] and MIPS CYGD [3] classify proteins according to many aspects, only one of them being the exact function exact (biochemical reaction catalyzed).

Correspondingly, there are several different machine learning settings an approaches to protein function prediction. Some works concentrate in predicting the top level of the taxonomies, in other words they aim to predict the main categories. For example, Lanckriet et al. [4] use kernel methods to combine multiple data sources to predict membership of yeast proteins in the 13 top level classes in the MIPS CYGD database [3]. Borgwardt et al. [5] use graph kernels to predict the 6 top level enzyme classes in the Enzyme Commission taxonomy. Finally, Cai et al. [6] predict membership in enzyme families one family at a time with support vector machines.

Our aim differs from the above approaches in that we are interested in predicting the membership of enzymes in the whole taxonomy. Thus the prediction problem is to output for each concept in the taxonomy whether the protein belongs to the concept or not. Our methods are so called structured output prediction methods, meaning that both learning and prediction happens simultaneously for the whole taxonomy. In this paper we concentrate in hierarchical taxonomies, although our methods generalize to general graph structures. In particular, we use EC hierarchy and the Gold Standard hierarchy [7]. In literature, the works that come close to our setting include the following. Clare and King [8] use decision trees to predict the membership in all classes in the MIPS taxonomy. Barutcuoglu et al. [9] combine Bayesian networks with a hierarchy of support vector machines to predict Gene Ontology, GO classification. Their work concentrate on the biological process sub-taxonomy of GO rather than the functional class. Blockeel et al. [10] use multilabel decision tree approaches to functional class classification according to the MIPS FunCat taxonomy.

We compare two kernel-based structured output prediction methods, Hierarchical Max-Margin Markov, HM^3 ^[11] and Maximum Margin Regression, MMR [12]. The former is a method specifically designed for hierarchical multilabel classification, the latter can be seen as a generalization of one-class support vector machine to structured output domains. As input features for these algorithms we use difference string kernel variants and the so called GTG features that can be seen as predicted conserved residues.

Polynomial and Gaussian kernels are used to construct higher-order features from the base kernels. We experiment with two datasets, a sample from KEGG LIGAND database (called EC dataset subsequently) and the recently introduced Gold Standard (GS) dataset [7].

## Results and discussion

### Comparing the polynomial and the Gaussian kernels

**Figure 2:**
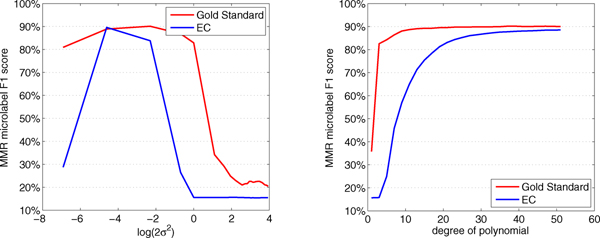
**Effect of kernel parameters to predictive accuracy**. Microlabel F1 scores using MMR methods for EC and Gold Standard datasets are depicted. In the figure on the left, we progressively increase the width of the Gaussian kernel. In the figure on the right the polynomial degree is progressively increased.

In preliminary tests with MMR we compared the polynomial and Gaussian kernels with varying kernel parameter values on top the GTG kernel. Increasing the degree of the polynomial kernel turned out to monotonically increase the accuracy, the increase being more slow and continuing further on the EC dataset than on the GS dataset. For both the Gaussian and polynomial kernels the best accuracy was almost the same (Figure 2). For subsequent experiments we chose the polynomial kernel due to its simplicity in interpretation in contrast to the Gaussian kernel.

### Results in EC class prediction

Here we report on experiments in predicting the EC-hierarchy with MMR and HM^3 ^using different sequence kernel combinations, with polynomial kernel applied on top. We run 5-fold cross-validation tests and report the 0/1 loss and the microlabel F1 score. A typical training run with MMR on this data took around 30 minutes. In contrast, HM^3 ^training time range was 1–24 hours, depending on the kernel. Our preliminary experiments indicated that GTG kernel is the only single kernel reaching microlabel F1 above 80%. Hence, in studying the kernel combinations we concentrated on augmenting GTG kernel with the different string kernels. Tables 1 and 2 shows the results of this comparison. As comparison we use a kernel nearest neighbor (NN): retrieving the training sequence *s*_*i *_with highest (sequence) kernel value *K*(*s*_*i*_, *t*) with the test sequence *t *and predicting the associated function *y*_*i *_of the training sequence.

**Table 1:** EC: F1 score over all microlabel predictions with different kernel combinations combined with linear or degree 51 polynomial kernel.

Sequence Kernel	Nearest neighbor (std)	MMR linear (std)	MMR poly-51 (std)	HN^3 ^poly-51 (std)
GTG	89.3	88.3 (0.9)	89.4 (0.8)	89.3 (0.8)
GTG+STR5	91.7	90.0 (0.5)	91.7 (0.4)	91.7 (0.4)
GTG+GAP611	90.9	86.0 (0.6)	90.9 (0.3)	90.9 (0.3)

**Table 2:** EC: 01-loss over all microlabel predictions with different kernel combinations combined with linear or degree 51 polynomial kernel.

Sequence Kernel	Nearest neighbor (std)	MMR linear (std)	MMR poly-51 (std)	HM^3 ^poly-51 (std)
GTG	16.8 (0.9)	18.6 (0.8)	16.7 (0.9)	16.7 (0.9)
GTG+STR5	14.2 (0.5)	16.9 (0.5)	14.2 (0.5)	14.2 (0.5)
GTG+GAP611	14.8 (0.6)	19.7 (0.6)	14.8 (0.5)	14.8 (0.5)

Overall predictive accuracy of all methods turns our to be very good. In all experiments, HM^3^, MMR and the nearest neighbor classifier are practically equal in accuracy, but only when polynomial kernel of high-degree (here *d *= 51) is used. MMR results with the linear kernel are clearly inferior and HM^3 ^turned out to perform even worse (data not shown). We notice that combining a string kernel (STR5) with GTG features is in most cases beneficial for all the methods. However, allowing gaps in subsequences (GAP611) does not seem to help.

### Results in Gold Standard prediction

In Gold Standard classification, GTG features turned out to be the only representation that had predictive value. String kernels and combinations of GTG and string kernels produced poor results. Consequently, we report here results with GTG features only.

In Tables 3 and 4 the microlabel F1 scores and 0/1 losses are reported. Here, HM^3 ^obtains the best microlabel F1 scores and MMR comes close second. For MMR, though, polynomial kernel is required to get the best performance. Nearest neighbor trails both the structured prediction methods, thus indicating that the structured prediction methods can utilize the superfamily information to obtain better predictions.

**Table 3:** Gold Standard: F1 score and standard deviation over all microlabel predictions with GTG kernel combined with linear or degree 51 polynomial kernel.

Sequence Kernel	Nearest neighbor (std)	MMR linear (std)	MMR poly-51 (std)	HM^3 ^linear (std)	HM^3 ^poly-51
GTG	88.0 (1.0)	81.9 (1.4)	89.3 (0.9)	90.2 (0.8)	89.6 (0.8)

**Table 4:** Gold Standard: 0/1-loss over all microlabel predictions with GTG kernel combined with linear or degree 51 polynomial kernel.

Sequence Kernel	Nearest neighbor (std)	MMR linear (std)	MMR poly-51 (std)	HM^3 ^linear (std)	HM^3 ^poly-51
GTG	24.1 (1.9)	36.3 (2.8)	21.4 (1.8)	23.3 (2.0)	21.6 (1.7)

### Effects of the nearest neighbor distance and the changing size of training set

In the final experiment, we aimed get some insight to when and why structured prediction methods work better than the nearest neighbor classifier. We wanted to check the effect of training set size with – the expectation being that small training set sizes would favor structured prediction as fewer close sequence neighbors were present. Also we wanted to check the effect of how similar sequence neighbor exists in the training set – the expectation being that nearest neighbor classifier would benefit from existence of close sequence neighbors.

**Figure 3:**
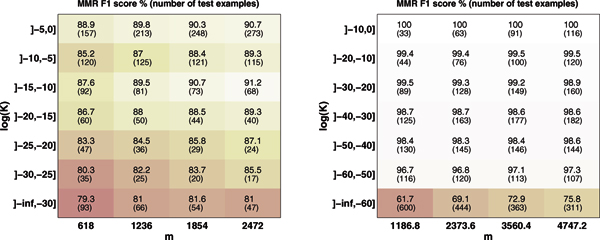
**Effect of training set size and nearest neigbor distance to MMR accuracy**. Depicted on the left is the MMR Gold Standard classification F1-score when trainingset size *m *and the kernel value between test example and trainingset nearest neighbor are increasing. On the right the same information is given for EC dataset. Values in parenthesis are average numbers of test examples with given training set size and kernel value interval.

**Figure 4:**
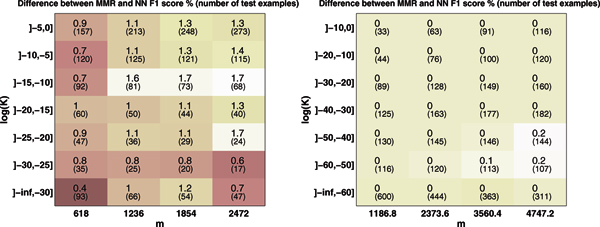
**The advantage of MMR over the NN classifier with different training set sizes and nearest neighbor distances**. Depicted on the left is the difference in MMR and NN Gold Standard classification F1-score when trainingset size *m *and the kernel value between test example and trainingset nearest neighbor are increasing on the GS dataset. On the right same information is given for the EC dataset.

Figure 3 depicts a heat map of MMR microlabel F1 score on the GS (left) and EC datasets (right) when the training set size is varied. The test set is divided along the vertical axis by the closest sequence neighbor in the dataset. It can be seen that on the GS data, the predictive accuracy improves by the increasing training set size, and the improvement is more clear for enzymes whose closest neighbor is at medium distance. On the EC dataset, the microlabel F1 is high even with small training set size and increasing the training set size affects the accuracy only a little, except for the enzymes with only distant neighbors. Figure 4 depicts a heat map of the difference in microlabel F1 score between MMR and NN. Red color indicates small difference, yellow indicates a larger difference in favor of MMR. On the GS dataset (Figure 4, left), both of the initial expectations turned out to be wrong: MMR works the better compared to NN the larger the training set and the closer are the nearest sequence neighbors. On the EC dataset (Figure 4, right), almost no difference can be seen irrespective of training set size or the closeness of sequence neighbors.

## Discussion

In machine learning it is well accepted that finding good input representations govern the learning performance much more than the particular learning algorithm that is being used. This view is reaffirmed in the experiments shown in this paper: irrespective of learning algorithm, good predictive accuracy depended on the use of the GTG features. Combination with polynomial kernel was useful for all sturcutred output methods and combination with string kernels had minor synergistic role in the case of EC dataset, and in fact a detrimental effect on the GS dataset.

Another main finding was that the ability of the structured output methods to overperform the simple nearest neighbor classifier is dependent on the output structure: with EC hierarchy the structured output methods at best could match the nearest neighbor accuracy. Moreover, this required the use of high degree polynomials in the input side, which means that the best performing input kernels were sparse and emphasizing large kernel values and can thus be interpreted as approximating the nearest neighbor classifier in a sense. In conclusion, it seems that the parent-child information contained in the EC hierarchy does not seem to aid function prediction.

An explanation for this may lie in the conceptualization in the EC hierarchy; it hierarchically divides the function space based on the properties of chemical reactions (e.g. types of bonds manipulated) not the properties of the enzymes (e.g. types of 3D folds). The GS hierarchy, on the other hand, is designed more from the point of view of enzyme evolution. The superfamily-family relations seem to aid the structured output methods in generalizing from the training data.

Another possible explanation for the good behavior of the nearest neighbor is a data quality issue. We speculate that many of the functions in the EC dataset may have been originally acquired via 'BLAST nearest neighbor' prediction, followed by wet lab verification. This approach obviously would miss any function not possessed by the nearest neighbor enzyme.

Overall, the predictive accuracy obtained in this paper is competitive with the state-of-the-art. For example Borgwardt et al. [5] report on 90.8% accuracy in predicting the top level membership of the EC hierarchy only, which is in the similar region as the microlabel F1 score obtained in this study, although their dataset was different. Note here that microlabel F1 contains prediction results of all nodes in the hierarchy, and is likely to be lower than top level accuracy. Elsewhere, Syed and Yona [13] report 89% accuracy in EC code prediction using a HMM based model, however, with a dataset restricted to 122 enzyme families with a large number of homologous sequences.

For research in structured output learning, it is noteworthy that MMR obtains the same level of accuracy as HM^3^, despite that MMR does not explicitly maximize the loss-scaled margins between the true output and competing outputs, the approach taken in most structured prediction methods. This difference makes MMR efficient learning approach, for example extensive parameter tuning is possible with MMR but starts to be tedious with loss-scaled margin maximization approaches even on medium-sized datasets.

## Conclusion

In this paper we have studied the utility of structured output prediction methods to enzyme function prediction. According to our experiments, structured output prediction is beneficial for predicting superfamily-family membership, but in predicting the EC classification, a nearest neighbor classifier does equally well. Overall predictive accuracy that is on par with the state-of-the-art results, is obtained by using the GTG sequence feature set and the polynomial kernel over the inputs.

## Methods

### Learning task

Our objective is to learn a function that, given (a feature representation) of a sequence, can predict (a feature representation) of an enzymatic reaction.

Learning algorithms that are designed for structured prediction tasks like the above, are many. We concentrate on kernel methods, that let us utilize high-dimensional feature spaces without computing the feature maps explicitly. Structured SVM [14], Max-Margin-Markov networks [15], Output Kernel Trees [16], and Maximum-Margin Regression (MMR) [12] are learning methods falling into this category. We consider a training set of (sequence, reaction)-pairs ${\left\{(x_{i},y_{i})|x_{i}\in X,y_{i}\in Y\right\}}_{i=1}^{m}$ drawn from an unknown joint distribution P(X,Y). A pair (*x*_*i*_, *y*), where *x*_*i *_is a input sequence and *y *∈ Y is arbitrary, is called a *pseudo-example *in order to denote the fact that the output may or may not have been generated by the distribution generating the training examples.

For sequences and reactions, respectively, we assume feature mappings *ϕ*: $X\mapsto F_{X}$ and *ψ*: $Y\mapsto F_{Y}$, mapping the input and output objects into associated inner product spaces $F_{X}$ and $F_{Y}$. The kernels *K*_*X*_(*x*, *x'*) = ⟨*ϕ*(*x*), *ϕ*(*x'*)⟩ and *K*_*Y*_(*y*, *y'*) = ⟨*ψ*(*y*), *ψ*(*y'*)⟩ defined by the feature maps are called the input and output kernel, respectively. Below, we discuss particular choices for the feature mappings and the kernels.

In structured prediction models based on kernels, the associations between the inputs and outputs are typically represented by a *joint *kernel, defined by some feature map joint for inputs and outputs. In this paper we use a joint feature map

$$\varphi (x,y):X\times Y\mapsto F_{X⊗Y},$$

where the joint map *φ*(*x*, *y*) = *ϕ*(*x*) ⊗ (*y*), is defined by the tensor product, thus consisting of all pairwise products *ϕ*_*j*_(*x*)*ψ*_*k*_(*y*) between inputs and output features. This choice gives us the joint kernel representation as elementwise product of the input and output kernels

*K*_*XY *_(*x*, *y*; *x'*, *y'*) = *K*_*X*_(*x*, *x'*)*K*_*Y *_(*y*, *y'*).

In this paper we apply the Hierarchical Max Margin Markov (HM^3^) [11] and Max Margin Regression [12] algorithms, the first being a structured prediction method specifically designed for hierarchical multilabel classification, and the latter being a very efficient generalization of one-class SVM to structured output spaces.

#### Hierarchical Max-Margin Markov algorithm

The Hierarchical Max-Margin Markov algorithm, HM^3 ^[11] is a variant of the Max-Margin Markov Network (M^3^N) structured output learning framework [15], tailored for hierarchical multilabel classification tasks. It learns a linear score function

*F*(*w*, *x*, *y*) = ⟨*w*, *φ*(*x*, *y*)⟩ = ⟨*w*, *ϕ*(*x*) ⊗ *ψ*(*y*)⟩

in the joint tensor product space. The model's prediction $\hat{y}$(*x*) corresponds to highest scoring output *y*:

$$\hat{y}(x)=argmax_{y}F(w,x,y).$$

As in most structured prediction frameworks, the criteria for learning the parameters *w *is to maximize the minimum loss-scaled margin

(1) *w*^*T*^(*φ*(*x*_*i*_, *y*_*i*_) - *φ*(*x*_*i*_, *y*)) - ℓ(*y*_*i*_, *y*)

over all pseudoexamples (*x*_*i*_, *y*). It is advisable to use a loss function that is smoothly increasing so that we can make a difference between 'nearly correct' and 'clearly incorrect' multilabel predictions. *Hamming loss*

$$\ell_{\Delta }(y,u)=\sum_{j}〚y_{j}\neq u_{j}〛,$$

has this property and is a typical first choice for its simplicity and ease of computation. For hierarchical classification, it is also possible to devise loss functions that are hierarchy-aware (c.f. [11,17-20]). In this paper, for simplicity and transparency, we resort to Hamming loss, however.

As with SVMs, to make the optimization problem solvable, there is a need to relax the margin constraints by allowing some slack. Allotting a slack variable *ξ*_*i *_for each example, the primal soft-margin optimization problem gets the form (c.f [11,14,15])

(2) $$\begin{array}{ll} \underset{w}{\min} & \frac{1}{2}{\left\|w\right\|}^{2}+C\sum_{i=1}^{m}\xi_{i}\\ \text{s}.\text{t}. & w^{T}(\varphi (x_{i},y_{i})-\varphi (x_{i},y))\geq \ell (x_{i},y)-\xi_{i},\forall i,y. \end{array}$$

and the corresponding Lagrangian dual is given by the quadratic programme

(3) $$\begin{array}{ll} \underset{\alpha \geq 0}{\min} & \sum_{i,y}\alpha (i,y)\ell (y_{i},y)-\frac{1}{2}\sum_{i,y}\sum_{j,y^{\prime }}\alpha (i,y)K(x_{i},y;x_{j},y^{\prime })\alpha (i,y^{\prime })\\ \text{s}.\text{t}. & \sum_{y}\alpha (i,y)\leq C,\forall i, \end{array}$$

where *K*(*i*, *y*; *j*, *y'*) = Δ*φ*(*i*, *y*)^*T *^Δ*φ*(*j*, *y'*), is the joint kernel defined on features

Δ*φ*(*i*, *y*) = *φ*(*x*_*i*_, *y*_*i*_) - *φ*(*xi*, *y*),

that is, joint feature difference vectors between the true (*y*_*i*_) and a competing output (*y*). Neither the primal nor the dual are amenable to solve with off-the-shelf QP solvers as both have exponential size in the output dimension, the primal has a large constraint set and the dual has correspondingly large dual variable set. There is a significant amount of research done on how to make optimizing the primal or the dual practical for realistic data sets [11,14,15,21]. HM^3^[11] is a marginal dual method (c.f. [15]), that translates the exponential-sized dual problem into an equivalent polynomially-sized form by considering the edge-marginals

(4) $$\mu (i,e,v)=\sum_{y\in Y}〚\psi_{e}(y)=v〛\alpha (i,y),$$

where *e *∈ *E *is an edge in the output hierarchy and *v *∈ {00, 01, 10, 11} is a possible labeling (class membership of either the parent node, the child node or both) for the edge.

Using the marginal dual representation, we can state the dual problem (3) in equivalent form as (for details, see [11]):

(5) $$\underset{\mu \in ℳ}{\max}\sum_{i,e,u}\mu (i,e,u)\ell (i,e,u)-\frac{1}{2}\sum_{i,j}\sum_{e\in E}\sum_{u,v}\mu (i,e,u)K_{e}(i,u;j,v)\mu (j,e,v),.$$

where ℳ denotes the marginal polytope, the set of all combinations of marginal variables (4) that have a counterpart in the dual feasible set in (3), and *K*_*e *_contains the joint kernel values pertaining to edge *e*.

This problem is a quadratic programme with a number of variables linear in both the size of the output hierarchy and the number of training examples. Thus, there is an exponential reduction in the number of dual variables from the original dual (3).

The marginal dual problem is solved by the conditional gradient algorithm (c.f. [22]) that iteratively the best feasible direction given the current gradient and uses line search to locate the optimal point in that direction. The feasible ascent directions turn out to correspond to pseudo-examples (*i*, *y*) that violate their margins (1) the most. Making use of the of hierarchical structure, the margin violators and consequently the feasible ascent directions are found in linear time by dynamic programming implementation of message-passing inference over the hierarchy [11].

#### Max Margin Regression algorithm

Like HM^3^, Max-Margin Regression (MMR) [12] also learns a linear function.

*F*(*w*, *x*, *y*) = ⟨*w*, *φ*(*x*, *y*)⟩

in the joint feature space given by the tensor product *φ*(*x*, *y*) = *ϕ*(*x*) ⊗ *ψ*(*y*). We note Szedmak et al. [12] define MMR with a bias term *b*, here we have adopted the equivalent convention that the bias term is subsumed into *ϕ *and *w*.

The main difference between the two algorithms is in the learning criterion. MMR aims to separate the training data *φ*(*x*_*i*_, *y*_*i*_) from the origin of the joint feature space with maximum margin, thus it can be seen analogous to the one-class SVM [23].

The primal form of the MMR optimization problem can be written as

(6) $$\begin{array}{ll} \min & \frac{1}{2}{\left\|w\right\|}^{2}+C\sum_{i}\xi_{i}\\ \text{s}.\text{t}. & \langle w,\varphi (x_{i},y_{i})\rangle \geq 1-\xi_{i}\\ & \begin{array}{cc} \xi_{i}\geq 0, & i=1,...,m. \end{array} \end{array}$$

The dual form of the MMR problem can be expressed as

(7) $$\begin{array}{ll} \max & \sum_{i=1}^{m}\alpha_{i}-\frac{1}{2}\sum_{i,j=1}^{m}\alpha_{i}\alpha_{j}K_{Y}(x_{i},x_{j})K_{Y}(y_{i},y_{j})\\ \text{s}.\text{t}. & \begin{array}{cc} 0\leq \alpha_{i}\leq C, & i=1,...,m. \end{array} \end{array}$$

It is noteworthy that the dimension of the output space does not affect the size of dual problem.

Another difference between MMR and most structured output prediction methods, including HM^3 ^is that there is no need to solve the loss-augmented inference problem (1) as part of the training. Although for hierarchies this problem can be solved in linear time, this is still a bottleneck in training the methods. MMR, due to its simple form, can be optimized with much faster algorithms. The present implementation uses the Augmented Lagrangian (c.f. [22]) algorithm.

### Data

In this paper, we use two datasets.

• **EC dataset **is a sample of 5934 enzymes from the KEGG LIGAND database [24]. The EC hierarchy to be predicted has four levels plus root and has size 1634 (1376 leaves, 258 internal nodes). In this version of the data, only single function per enzyme is reported.

• **Gold Standard **dataset contains 3090 proteins which are classified into superfamily and family classes by their function [7]. The hierarchy to be predicted has two levels plus root and has size 493 (487 families, 5 superfamilies).

#### Input feature representations

Kernels for sequences have been actively developed during recent years [25-27]. We selected the following representations for these trials:

**Substring spectrum kernels **[25] are sometimes referred simply to as string kernels. They induce a feature space where each substring of predefined length *p *is allocated a dimension, and the feature values are counts of these feature values. In the experimental section we refer as STR*p *the length-*p *substring spectrum kernel. The string kernels can be computed in linear time via the use of suffix trees or suffix arrays [28].

**Gaps or mismatches **[26] can be allowed in substring occurrences. Gaps can be be restricted in number, length or both, or long gaps can be penalized by down-weighting [25]. In addition, gaps can be restricted to certain positions of the substring. In our experiment we refer as *GAPxyz *a kernel defined on length-*x *substrings where at most *y *mismatches (out of *x*) of length at most *z *is allowed.

Gappy substring kernels take generally a quadratic time in the length of the compared sequences to compute [25].

**GTG kernel**. The so called Alignment Trace Graph [29,30] is an approach to find residues that potentially are well conserved and thus may be a part of the active center. The GTG (Global Trace Graph) kernel obtained from this method is defined on features *ϕ*_*AA*, *C*_(*s*) = 1 denoting a (potentially conserved) residue of type *AA *in cluster *C *(potential location within active center) in sequence *s*.

The GTG representation comes as explicit sparse feature vectors.

A benefit of kernel methods is that in dual representation, features can be combined without significant extra cost. The polynomial kernel

*K*_*poly*_(*x*, *x'*) = (*K*(*x*, *x'*) + *c*)^*d*^,

efficiently computes a *d*-degree polynomial features out of the original features, in time linear in the kernel matrix size. Thus, working in high-dimensional feature spaces becomes computationally feasible. In the case of the above base kernels, the polynomial feature space consists of occurrences of all combinations of up to *d *subsequences (in the case of string kernels) or conserved residues (in the case of the GTG kernel). The Gaussian kernel

$$K_{Gaussian}(x,x^{\prime })=\exp\left(-\frac{{\left\|x-x\right\|}^{2}}{2\sigma^{2}}\right)$$

can be seen as the infinite dimensional polynomial kernel, with high polynomial degree terms exponentially down-weighted. The width of the Gaussian *s *corresponds to the degree of the polynomial, small values of *σ *corresponding to high-degree *δ *[31].

#### Output feature representation

**Figure 5:**
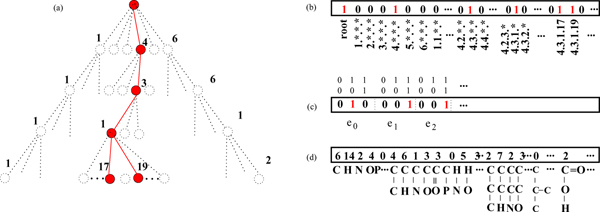
**Representations of enzyme function**. Schematic representation of enzymatic reaction in the EC-hierarchy is shown in (a). Different feature representations are depicted: node indicators (b), edge labeling indicators (c), and reaction kernel (d).

For representing hierarchies, the MMR algorithm and HM^3 ^use different encodings. HM^3 ^uses edge labeling indicators (Fig. 5c)

$$\phi_{e,y_{e}}(\rho )=〚\text{given }\rho ,e\text{ should be labeled as }y_{e}〛$$

The benefit of this representation is in that dependencies between parent and child can be encoded in the feature map, which may ease learning correlations between the inputs and outputs.

In MMR, it is possible also to use node indicators (Fig. 5b)

*ϕ*_*v*_(*ρ*) = ⟦*ρ *belongs to node *v*⟧,

that simply state whether given node is part of the multilabel or not. This representation does not contain any information of the hierarchy, however. Feature embedding can be made hierarchy-specific by replacing the indicators with real valued functions that depend on the location in the hierarchy. For example,

*ϕ*_*v*_(*y*) = *γ*^-1 ^⟦*ρ *belongs to node *v*⟧,

where *γ *> 1 and *d *is the depth of the node, will emphasize the importance of nodes deep in the hierarchy, and thus concentrate the learning algorithms effort to getting the difficult deep nodes correct. In our experiments with the MMR algorithm we use this embedding with *γ *= 10.

As MMR is not tied to hierarchical outputs, in principle it would be possible to use any kernel on the enzyme function. In Fig. 5d), one alternative, a subgraph spectrum of the reactant molecule set is depicted.

### Measuring success of prediction

We use two measures to characterize the performance of the compared learning approaches

• Zero-One loss is the proportion of examples for which the predicted labeling (vector) is incorrect: $\ell_{0/1}=\frac{1}{m}\sum_{i}〚{\hat{y}}_{i}\neq y_{i}〛$

• Microlabel F1 score is obtained by pooling together all individual predictions ${\hat{y}}_{ij}$ of labels of node *j *∈ *V *in example *x*_*i*_, *i *= 1,..., *m*, computing the precision $Prec=\frac{TP}{TP+FP}$ and recall $Rec=\frac{TP}{TP+FN}$ where *TP*, *FP*, *FN *denote the number of true positive, false positive and false negative predictions in the pool. Microlabel F1 is then given by

$$F_{1}=\frac{2Pr}{Pr+Rec}.$$

## Competing interests

The authors declare that they have no competing interests.

## Authors' contributions

JR and SS are the main architects of the machine learning methods. JR and KA have designed the experiments and written the article. KA has participated in developing the methods and has conducted the experiments. LH has participated in developing the research ideas and contributed the GTG data. EP has participated in development of the methods and preparing the experiments.
